# Prevalence of *Onchocerca lupi* in shelter dogs from an endemic region of the Southwestern USA

**DOI:** 10.1186/s13071-025-06988-5

**Published:** 2025-08-05

**Authors:** Maureen A. Kelly, Erin Clarke, Hassan Hakimi, Christine M. Budke, Guilherme G. Verocai

**Affiliations:** 1 Department of Veterinary Pathobiology, College of Veterinary Medicine and Biomedical Sciences, Texas A&M University, College Station, TX 77843 USA; 2 Albuquerque Animal Welfare, Albuquerque, NM 87112 USA; 3 Department of Veterinary Integrative Biosciences, College of Veterinary Medicine and Biomedical Sciences, Texas A&M University, College Station, TX 77843 USA

**Keywords:** Canine ocular onchocercosis, Filarioid nematodes, Molecular diagnostics, *Onchocerca lupi*, Real-time PCR, Vector-borne diseases, Zoonotic onchocerciasis

## Abstract

**Background:**

*Onchocerca lupi* is a zoonotic, vector-borne filarioid nematode that mainly infects wild and domestic canids in the Southwestern USA, Europe, Asia, and Africa. Clinical canine infections are associated with ocular disease, characterized by the presence of nodules and conjunctivitis. Subclinical cases can be challenging to diagnose, even with evaluation of cutaneous tissues for microfilariae. Current diagnostic tests include conventional polymerase chain reaction (cPCR) to detect *O. lupi* DNA, and, alternatively, real-time PCR (qPCR), which provides more rapid results and higher throughput. The objectives of this study were to: I) optimize a novel qPCR assay that detects *O. lupi* and II) to assess the prevalence of *O. lupi* in shelter dogs from Albuquerque, NM, USA.

**Methods:**

This probe-based qPCR was optimized with a detection threshold of 0.33 pg for DNA of an adult female *O. lupi*. We further optimized the assay by performing a dynamic range test to determine the ideal dilution factor and inclusion of an internal positive control. We collected skin snips from the interscapular region of 404 dogs between January and September 2023. Demographics were recorded, including age, sex, American Kennel Club breed groups, and coat color. Dogs were separated into age groups, including juveniles ≤ 1 year old (*n = *120; 29.7%), adults > 1–7 years old (*n = *260; 64.3%), and seniors > 7 years old (*n = *24; 5.9%). Of those, 194 were female, and 210 were male. We also had nine different American Kennel Club breed groups represented, as well as two coat colors: single (33.0%) and mixed (67.0%). Genomic DNA was subjected to cPCR followed by Sanger sequencing and our probe-based qPCR. Both PCRs targeted a fragment of the cytochrome oxidase c subunit 1 (*cox1*) of the mitochondrial DNA. We performed statistical analysis to assess any association between exposure factors, such as age, sex, breed, and coat color and the outcome, whether *O. lupi* was present.

**Results:**

Overall, eight (1.9%; 95% confidence interval (CI) 0.8–3.8%) dogs tested *O. lupi*-positive via qPCR and five (1.2%; 95% CI 0.4–2.8%) via cPCR. Of the qPCR-positive dogs, six were adults and two were juveniles. Age (*P* = 0.704), sex (*P* = 0.910), breed groups (*P* = 0.217), and coat color (*P* = 0.781) were not statistically associated with a qPCR-positive result with a cutoff of *P* < 0.2. In addition, 20 dogs tested positive for *Cercopithifilaria bainae* via cPCR and sequencing, but these did not cross-react with our qPCR.

**Conclusions:**

This is the first epidemiological study on *O. lupi* in a canine population from an urban center within an endemic area in North America. Active surveillance using reliable diagnostic tools can better elucidate the epidemiology of this zoonotic parasite and enable the implementation of strategies for control and prevention.

**Graphical Abstract:**

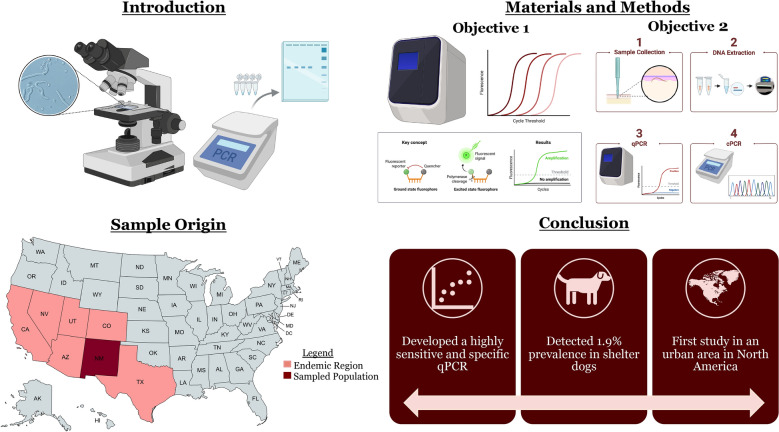

**Supplementary Information:**

The online version contains supplementary material available at 10.1186/s13071-025-06988-5.

## Background

*Onchocerca lupi* is an emerging, zoonotic filarioid nematode that is known to infect dogs, cats, wild canids, and humans [1–10]. It has been documented in various regions, including Europe, Africa, Asia, and the Southwestern USA [2–4, 11–18]. The clinical signs caused by *O. lupi* can vary on the basis of disease progression [19]. In the acute stage, animals may present with ocular discharge and conjunctivitis. The chronic stage may involve the development of nodules within the conjunctiva, sclera, and retrobulbar space [3, 18, 20, 21]. However, many cases of infection are subclinical and will likely go undiagnosed.

Various aspects of the biology of *O. lupi* remain unknown, including life cycle parameters in the definitive host and the range of suitable dipteran intermediate hosts across endemic areas on different continents. As of now, *O. lupi* DNA has been found in blackflies (*Simulium tribulatum*) and biting midges (*Culicoides* spp.) in the Southwestern USA [4, 22]. In addition, *O. lupi* DNA has been isolated from the heads and bodies of *Simulium griseum* [18]. Another factor to consider is that there are no commercially available diagnostic tests for *O. lupi*. Currently, a commonly used diagnostic test for detecting *O. lupi* infection in dogs is microscopic examination of saline sedimentation from multiple skin snips taken from different anatomic locations, such as the nose, forehead, and interscapular region [18, 23–25]. A more sensitive and common diagnostic test is conventional polymerase chain reaction (cPCR), a molecular technique capable of detecting small amounts of parasite DNA in clinical samples, even when microfilariae may not be detected through microscopy [4, 18, 23–25]. This creates the need for accurate, rapid diagnostic tests that can be used to detect infection in animals and humans. Existing commercially available point-of-care tests for common companion animal parasites, such as *Dirofilaria immitis*, have not shown cross-reactivity with *O. lupi* [26]. There are a few molecular-, serological-, and imaging-based diagnostic tests that have been developed for *O. lupi*, including probe-based quantitative real-time PCR (qPCR), indirect enzyme-linked immunosorbent assay (ELISA), and ultrasound testing [27–33]. There is a need for a highly sensitive and specific qPCR with the incorporation of an internal positive control that would allow for the determination of potential PCR inhibition.

With the development of new molecular tools, additional epidemiological studies can be completed to determine the prevalence of *O. lupi* in known endemic regions and potential establishment in new areas. In this study, we optimized a probe-based qPCR assay for *O. lupi*. We used this modified qPCR to assess the prevalence of *O. lupi* in shelter dogs and to evaluate potential risk factors associated with infection.

## Methods

### Probe and primers optimization for qPCR

Probe and primers targeting the mitochondrial cytochrome c oxidase subunit 1 (*cox1*) were modified from previously described work that was designed to detect DNA of *O. lupi* in definitive hosts and putative vector samples [27]. After preliminary testing and validation of the original protocol, a few modifications were implemented. Briefly, we adapted the forward primer by changing a single base pair to ensure accurate detection of the *O. lupi* genetic lineage found in the USA (O.l.F 5′-GGAGGCGGTCCTGGTAGTAG-3′; O.l.R 5′-GCAAACCCAAAACTATAGTATCC-3′) and TaqMan^®^ hydrolysis probe (FAM-5′-CTTAGAGTAGAGGGTCAGCC-3′-non-fluorescent quencher-MGB) [27]. This adaptation was made on the basis of DNA sequences available in GenBank originating from various locations within the USA, which were consistently identical [3, 4, 17, 19, 20, 22, 34, 35] (Supplementary Data, Supplementary Fig. S1). Adult nematode specimens preserved in 70% ethanol that were surgically removed from ocular tissue of naturally infected dogs and morphologically confirmed as *O. lupi* were utilized as positive controls. Specimens were subsequently subjected to enzymatic and mechanical disruption to ensure that the tissue was effectively lysed. Prior to DNA extraction, specimens were placed overnight in a thermomixer (Eppendorf, Germany) at 56 °C with a movement rate of 350 RPM. Genomic DNA from adult *O. lupi* specimens was extracted using the DNeasy^®^ Blood and Tissue Kit (Qiagen, Valencia, CA, USA) following the manufacturer’s instructions. DNA extractions were kept frozen at −20 °C until further use. Then, we performed a standard curve using a tenfold serial dilution of adult worm DNA of *O. lupi*. Next, we performed a dynamic range test using skin samples from dogs that were negative for filarioid nematode DNA via cPCR and qPCR. These samples were then spiked with *O. lupi* adult worm DNA at a concentration of 0.657 ng/μL to determine the ideal concentration range. We then completed a second dynamic range test that utilized three dogs’ skin samples that were positive via cPCR and qPCR using only the first three dilution factors that had a positive cycle threshold (CT) value.

In addition, we tested specimens of filarioid nematodes from three different species known to occur in dogs in the USA to ensure that our optimized qPCR did not exhibit cross-reactivity. These additional samples included DNA of an adult *D. immitis* specimen, *D. immitis* microfilariae in canine whole blood, *Acanthocheilonema reconditum* microfilariae in canine whole blood, and *Cercopithifilaria bainae* microfilariae in a canine skin sample.

### Epidemiological study location

Shelter dogs were sampled from Albuquerque, NM, Southwestern USA, from January to September 2023. The city of Albuquerque is located in Bernalillo County, where there is a warm temperate semi-desert climate [36]. Bernalillo County has an elevation of 4967 ft and is one of the largest counties in the USA, with a total area of 187.2 mi^2^ [37, 38]. According to the US Census Bureau, the 2023 population of Albuquerque was estimated at 560,274 [37]. To be included in this study, dogs must officially belong to the selected shelter, have an estimated age of 6 months or older, and have received either a spay or neuter surgery while residing at the shelter. Skin samples were collected prior to the spay or neuter being performed. This allowed for sample collection from the interscapular region to occur prior to surgery while under general anesthesia. Dogs that were included in this study had one or two superficial skin samples collected. Demographic information recorded during collection included age, sex, American Kennel Club (AKC) breed groups, and coat color [39].

### Sample size calculation

Since the prevalence of *O. lupi* within the study population was unknown, we set the estimated true proportion (*P*) at 50% (0.5) and our desired precision (*e*) at 5% (0.05) and used a 95% confidence level (*Z* = 1.96). The sample size was calculated using the following formula assuming a perfect test.

$$n= \frac{\left({Z}^{2} x P x (1-P)\right)}{{e}^{2}}$$

The required sample size was determined to be 385 individuals. However, we intentionally enrolled an additional 19 individuals, respecting all sampling criteria, to account for incomplete or insufficient skin sample collection.

### DNA extraction

Skin samples were received in a 1.5-mL microcentrifuge tube (Eppendorf, Germany) in 70% ethanol. Once received and transferred to a new 1.5-mL microcentrifuge tube, skin samples were processed to ensure that all ethanol was evaporated using a fast, efficient vacuum centrifuge (Vucufuge^®^ Plus, Eppendorf, Germany). Skin samples were enzymatically and mechanically disrupted to ensure the tissue was lysed. Prior to extraction, samples were placed in a thermomixer (Eppendorf, Germany) overnight at 56 °C with a movement rate of 350 RPM. The following day, genomic DNA was extracted using a compact, automated nucleic acid purification platform using a tissue kit following the manufacturer’s instructions (Maxwell^®^ RSC 48, Promega Corporation, Madison, WI, USA). All extracted DNA was stored at −20 °C until further analysis.

### cPCR

Conventional PCR (cPCR) was used to detect filarial nematode DNA using pan-filarial primers also targeting a fragment of approximately 635 base pairs (bp) of the *cox1* region (COIintF: 5′-TGATTGGTGGTTTTGGTAA-3′ and COIintR: 5′-ATAAGTACGAGTATCAATATC-3′) [40]. All reactions were performed in a 25-µL reaction containing 8.75 µL of molecular-grade water, 0.625 µL (10 µM) per primer, 12.5 µL of 2 × GoTaq^®^ Green Master Mix (Promega Corporation, Madison, WI, USA), and 2.5 µL of DNA template. Cycling conditions consisted of 95 °C for 2 min, followed by 40 cycles at 95 °C for 45 s, 52 °C for 45 s, and 72 °C for 90 s, with a final extension step at 72 °C for 5 min. All runs included positive and negative controls. The positive control was DNA from an adult *Setaria equina* specimen, and the negative control was molecular-grade water. PCR products were subjected to a 1% agarose gel stained with GelRed^™^ Nucleic Acid Gel Stain (GoldBio, St. Louis, MO, USA). The agarose gel was visualized using ultraviolet (UV) light to determine whether amplicons were present (ChemiDoc Imaging System, BioRad, Hercules, CA, USA). All cPCR products that showed an amplification band were purified using a Cycle Pure E.Z.N.A.^®^ Kit (Omega Bio-Tek, Norcross, GA, USA) following the manufacturer’s instructions. Purified products were sent for Sanger sequencing using the original cPCR primers (Eurofins, Louisville, Kentucky, USA). Then, forward and reverse sequences were generated and compared with genetic data available in GenBank using the BLAST function and aligned for phylogenetic analysis using Molecular Evolutionary Genetics Analysis (MEGA) X software [41].

### Probe-based qPCR

All samples were subjected to the optimized probe-based qPCR that utilized the ideal dilution factor (1:5) for skin samples. We incorporated the use of an internal positive control, VetMAX^™^ Xeno^™^, to help detect PCR inhibition [42, 43]. To achieve this, we first determined whether a higher annealing temperature would still enable DNA detection outside the universal cycling conditions (ThermoFisher Scientific Inc., Waltham, Massachusetts, USA). Once this was completed, we then performed a dynamic range test to find the ideal concentration of synthetic DNA when used in combination with target DNA (*O. lupi*). Once this was completed, we used DNA extracted from a canine skin snip spiked with the internal positive control, VetMAX Xeno DNA (ThermoFisher Scientific Inc., Waltham, Massachusetts, USA), to ensure that inhibition was not occurring. The TaqMan^®^ probe and primers described above were utilized. All reactions consisted of 1.5 µL of molecular-grade water, 0.5 µL (50 µM) of each primer, 0.5 µL (20 µM) of probe, 10 µL 2 × of TaqMan^®^ Fast Advance Master Mix (Applied Biosystems, Waltham, MA, USA), 1 µL of VetMAX^™^ Xeno^™^ Internal Positive Control—VIC^™^ Assay (ThermoFisher Scientific Inc., Waltham, Massachusetts, USA), 1 µL of VetMAX^™^ Xeno^™^ Internal Positive Control DNA (ThermoFisher Scientific Inc., Waltham, Massachusetts, USA), and 5 µL of DNA template in a 20-µL reaction volume. All qPCR assays were performed on a QuantStudio^™^ 3 real-time PCR system (Applied Biosystems, Waltham, MA, USA). Cycling conditions included an initial denaturation stage of 95 °C for 3 min to allow for DNA separation, followed by 40 cycles of a two-step PCR stage at 95 °C for 10 s and 64 °C for 30 s. All runs included two positive controls and a single negative control. One positive control consisted of extracted DNA from an adult *O. lupi* specimen, which was confirmed morphologically and molecularly. The second positive control consisted of diluted VetMAX^™^ Xeno^™^ DNA (10^–2.5^). Nuclease-free molecular-grade water was used as a negative control. The results of the qPCR were analyzed using Design & Analysis 2 software (Applied Biosystems, Waltham, MA, USA) to determine the cycle threshold (CT) value for each sample.

### Statistical analysis

Data were analyzed using STATA^®^ version 18.0 BE-Basic Edition (College Station, TX, USA). We measured the frequency of each demographic variable and the results of both diagnostic techniques. The chi-squared test was used to evaluate possible associations between demographic exposure variables (e.g., age, sex, AKC breed group, and coat color) and the outcome variable (*O. lupi* detection). A cutoff of *P* ≤ 0.2 was used to determine statistical significance. This level of significance was selected because this study is considered exploratory, which will allow for future investigations into potential trends in variables that were significant.

## Results

### qPCR optimization and validation

**Fig. 1:**
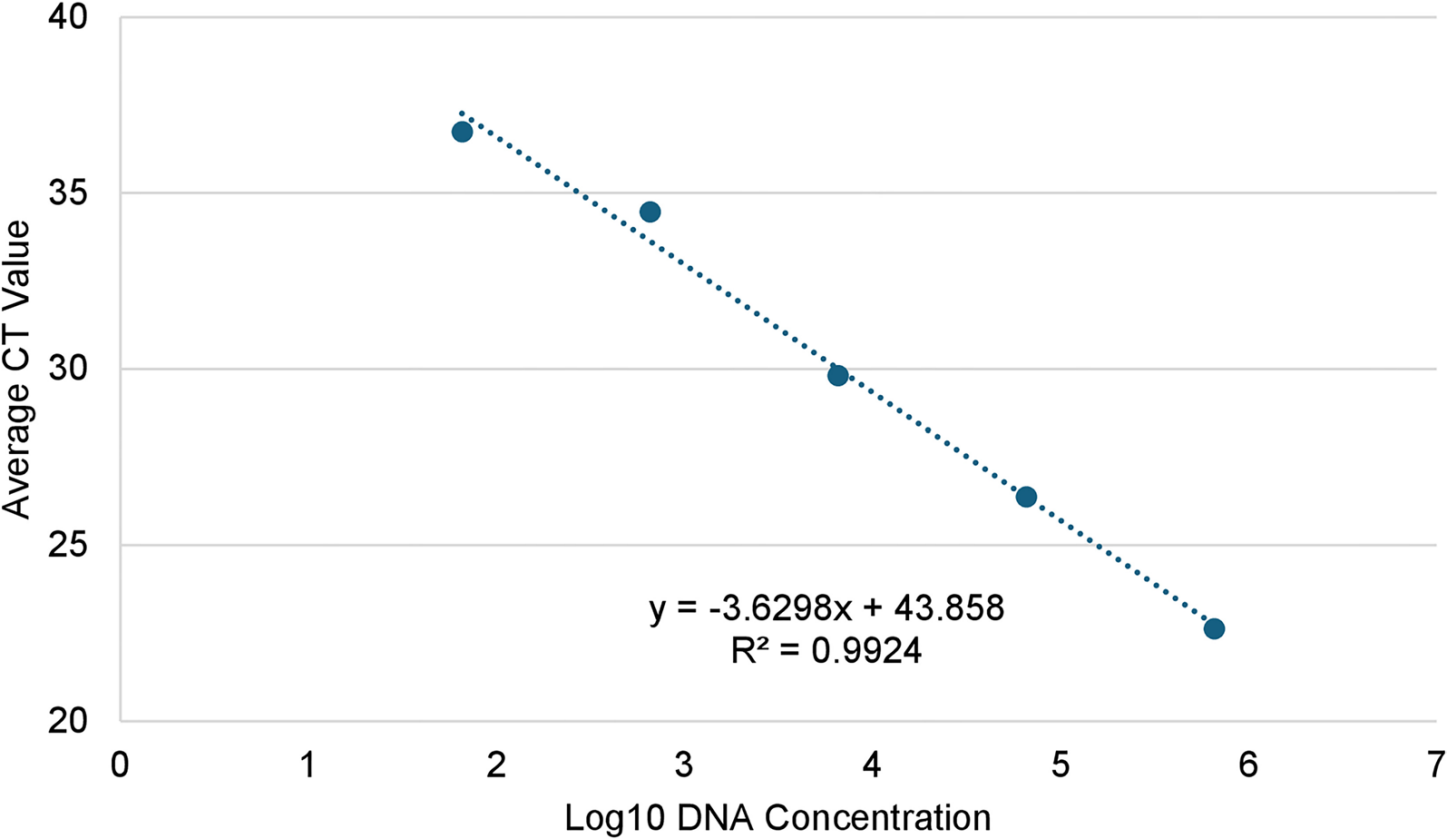
Standard curve of qPCR for the detection of *Onchocerca lupi*. This was created by plotting the average CT values of triplicates using a ten-fold serial dilution of adult worm parasite genomic DNA

**Fig. 2:**
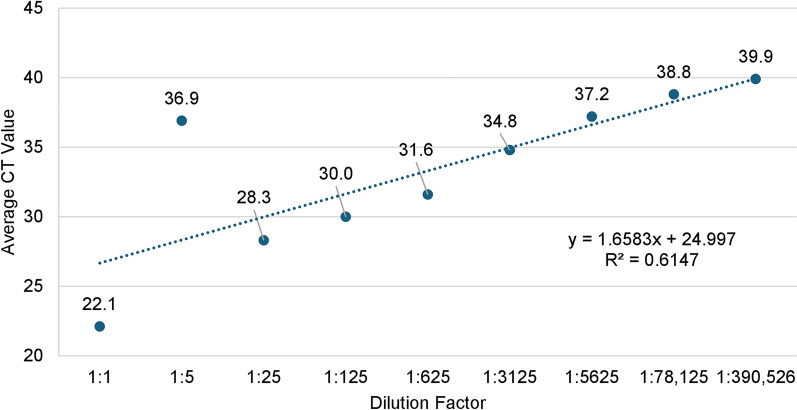
Dynamic range test of skin snip from a single dog that was negative in cPCR spiked with adult worm DNA of *Onchocerca lupi*. This curve was plotted by taking the average CT of triplicates using a five-fold dilution factor

**Fig. 3:**
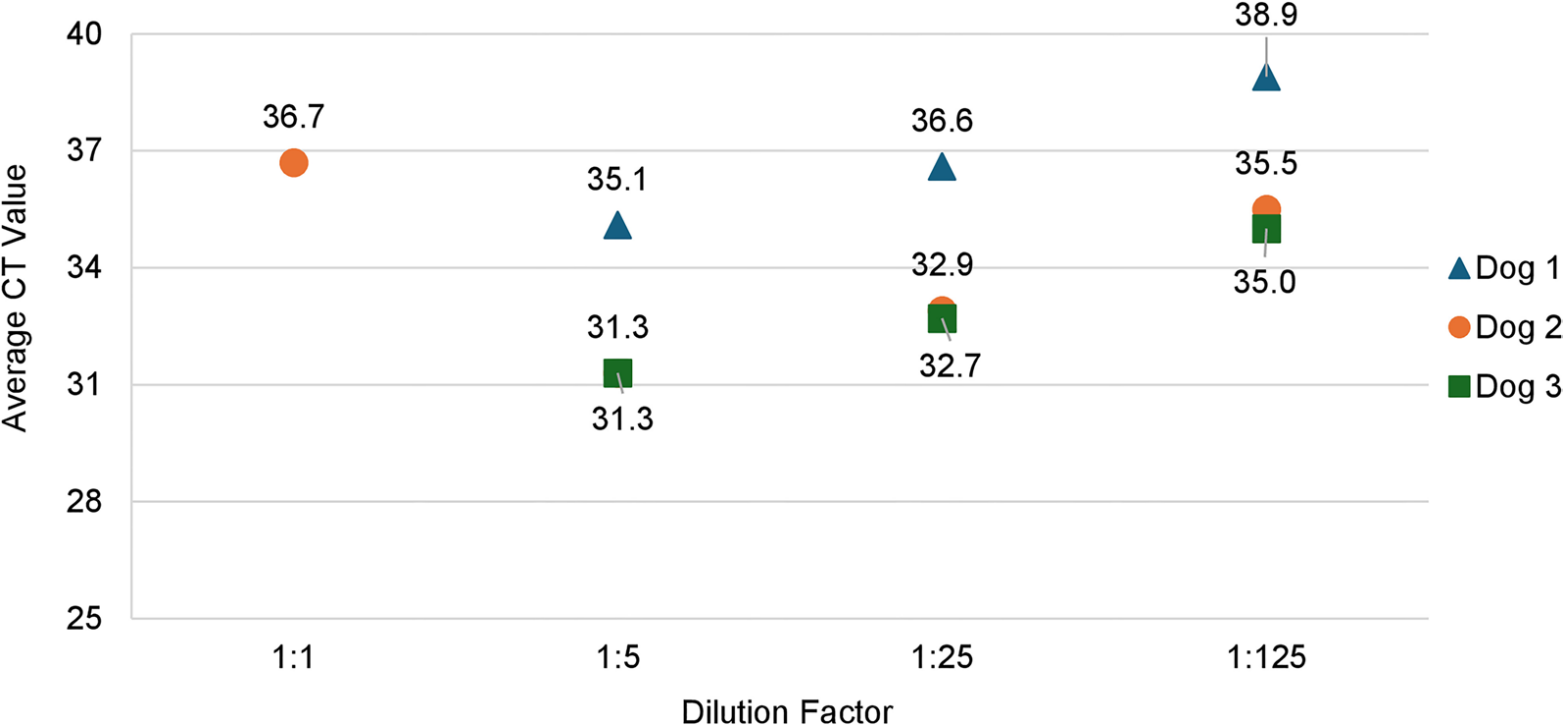
Dynamic range test of skin samples from three dogs that were positive in cPCR and qPCR for *Onchocerca lupi*. This curve was plotted by taking the average CT of triplicates using a five-fold dilution factor

Initially, when utilizing the previously published qPCR protocol [27], 78.9% (*n = *319/404) of dogs were screened, detecting a late amplification with a CT value of 39.9 in a single dog. This CT value did not fall within the acceptable range of detection for the original protocol and was not considered positive [27]. Simultaneously, we screened the same set of dog samples via cPCR and found that four dogs were confirmed positive for *O. lupi* using Sanger sequencing results. This led to the modification of the original protocol to include the change of a single base pair (T to C) in the forward primer to allow for an accurate detection of the single genetic lineage ever found in the USA [3, 4, 17, 20–22, 24, 34, 44] (Supplementary Data, Supplementary Fig. S1). The limit of detection (LOD) for *O. lupi* using this qPCR was 0.33 pg of parasite DNA. The novel qPCR assay exhibited excellent linearity between the DNA concentration and CT values over six orders of magnitude (Fig. 1). The efficiency of the qPCR was 89.0%, and the coefficient of correlation (*R*^2^) was 99.2% (Fig. 1). In the first dynamic test, we utilized DNA extracted from a skin snip of a *O. lupi*-negative, laboratory, purposed-bred Beagle that was spiked with adult nematode DNA (0.657 ng/µL) and showed the ideal range of dilution to be between 1:5 and 1:125 (Fig. 2). In the second dynamic range test, we utilized three skin samples from dogs that were positive via cPCR for *O. lupi* (Fig. 3). In the 1:1 dilution factor, only a single sample amplified. However, all three samples amplified in the dilution factors that followed (1:5–1:25). On the basis of the CT values, we selected a 1:5 dilution of skin sample DNA to ensure detection and optimal amplification of *O. lupi* DNA (Fig. 3). The ideal number of copies for using an internal positive control (Vet MAX^™^ Xeno^™^, ThermoFisher Scientific Inc., Waltham, MA, USA) was 4000 copies/μL (Supplementary Data, Supplementary Fig. S2).

### Epidemiological study

We sampled skin snips from 404 dogs from Albuquerque, NM, located in the Southwestern USA. Of these, two skin samples were collected from 98.7% of dogs (*n = *399/404), and 1.2% (*n = *5/404) had a single skin snip collected. Most dogs (*n = *260/404, 64.3%) were considered adults (i.e., > 1–7 years old). Of the enrolled dogs, 52% were male, and 48% were female. On the basis of the AKC breed groups, the top three groups represented were Terrier (*n = *102/404; 25.2%), Toy (*n = *92/404; 22.9%), and Herding (*n = *88/404; 21.7%). The majority of dogs sampled had a coat with multiple colors (67%), compared with those with a single-color coat (33%) (Table 1).

**Table 1:** Demographics of dog population sample from Albuquerque, NM, USA (*n = *404)

Demographics	No. (%)		
Age			
Juvenile (≤ 1 year old)	120 (29.7)		
Adult (> 1–7 years old)	260 (64.3)		
Senior (> 7 years old)	24 (5.9)		
Sex			
Female	194 (48.0)		
Male	210 (52.0)		
AKC breed groups			
Herding group		88 (21.7)	
Hound group		16 (3.9)	
Non-sporting group		32 (7.9)	
Sporting group		18 (4.4)	
Terrier group		102 (25.2)	
Toy group		92 (22.9)	
Working group		42 (10.4)	
Miscellaneous		12 (3.0)	
Unknown		2 (0.5)	
Coat color			
Single color			133 (33.0)
Multiple colors			271 (67.0)

Overall, we detected *O. lupi* DNA in skin snips from eight dogs (1.9%; 95% confidence interval (CI) 0.8–3.8%) using this probe-based qPCR compared with five dogs (1.2%; 95% CI 0.4–2.8%) that were confirmed via Sanger sequencing results from the cPCR (Table 2). Of the eight dogs that were confirmed positive for *O. lupi* via qPCR, two were juveniles (6 months to < 1 year old), and six were adults (1–7 years old). No senior dogs were found to be positive for *O. lupi*. The cPCR followed by Sanger sequencing only detected 62.5% (*n = *5/8) of positive dogs, these included two juveniles and three adults. All ten sequences were accessioned in GenBank (PV764666–75) and were 100% identical to each other and to all homologous *O. lupi* sequences available from the USA. The remaining samples (*n = *3/8) yielded inconclusive sequencing results. It is worth highlighting that an additional 20 dogs (4.9%) were positive via cPCR for *C. bainae*. However, these samples did not amplify via the novel qPCR, confirming the specificity of this modified assay.

**Table 2:** Summary of results of probe-based qPCR and conventional PCR for *Onchocerca lupi*

	cPCR positive	cPCR inconclusive	cPCR negative	Total
qPCR positive	5	3	0	8
qPCR negative	20	0	376	396
Total	25	3	376	404

The results of the univariate analysis of risk factors are presented in Table 3. Age (*P* = 0.704), sex (*P* = 0.910), AKC breed group (*P* = 0.217), and coat color (*P* = 0.781) were not statistically associated with a qPCR positive result for *O. lupi*.

**Table 3:** Risk factors associated with prevalence of *Onchocerca lupi* among dogs in Albuquerque, NM, USA

Variable	Total no.	*Onchocerca lupi*		
		No. (%)	95% CI	Chi-squared *df* *P*-value
Age				
Juvenile (≤ 1 year old)	120	2 (1.6)	0.2–5.8	*χ*^2^ = 0.702 *df *= 2 *P* = 0.704
Adult (> 1–7 years old)	260	6 (2.3)	0.8–4.9	
Senior (> 7 years old)	24	0	–	
Sex				
Female	194	4 (2.0)	0.5–5.1	*χ*^2^ = 0.012 *df* = 1 *P* = 0.910
Male	210	4 (1.9)	0.5–4.8	
American Kennel Club breed group				
Herding group	88	2 (2.2)	0.2–7.9	*χ*^2^ = 10.735 *df* = 8 *P* = 0.217
Hound group	16	0	–	
Non-sporting group	32	0	–	
Sporting group	18	2 (11.1)	1.3–34.7	
Terrier group	102	3 (2.9)	0.6–8.3	
Toy group	92	1 (1.0)	0.02–5.9	
Working group	42	0	–	
Miscellaneous	12	0	–	
Unknown	2	0	–	
Coat color				
Single color	133	3 (2.2)	0.4–6.4	*χ*^2^ = 0.0775 *df* = 1 *P* = 0.781
Multiple colors	271	5 (1.8)	0.6–4.2	

*df*, degrees of freedom; CI, confidence interval

## Discussion

This is the first epidemiological study on *O. lupi* performed in an urban center within an endemic area of North America. We used an optimized probe-based qPCR to detect *O. lupi* infections within skin samples of shelter dogs. Overall, we determined a 1.9% prevalence using this qPCR, compared with a 1.2% prevalence found by cPCR. It is essential to note that the nearly 2% prevalence may be an underestimation owing to biological and methodological reasons. As molecular detection of *O. lupi* DNA in skin snips targets the microfilariae stage in dermal tissues, non-patent infections could not be detected. In addition, the non-homogeneous microfilaridermia across anatomic locations of the host’s body and its periodicity could have further contributed [25].

The Southwestern USA is an endemic area for *O. lupi*; however, previous reports have come from client-owned dogs presenting with clinical disease [3, 4, 19, 21, 22]. Our study was the first to assess the prevalence of this parasite in a shelter dog population in the USA, enrolling dogs regardless of clinical suspicion of ocular onchocercosis. Moreover, our study focused on dogs from an urban setting, suggesting local parasite transmission. In addition to the Southwestern USA, *O. lupi* cases have been reported in dogs from other regions of North America. Specifically, two cases from Florida, two cases in Minnesota, a single case in New York, and two cases from Canada, all confirmed morphologically and molecularly [19, 24, 33, 35]. When available in these published case reports, the history of such cases can be traced back to the Southwestern USA.

There has been only a single study looking at the prevalence of *O. lupi* in North America, specifically in companion animals owned by Navajo populations in Arizona, New Mexico, and Utah [4]. That study found a 9.4% prevalence combining samples from both dogs and cats, and, similar to the present study, did not target animals with clinical onchocercosis [4]. One may infer that most *O. lupi*-positive animals were dogs, since, to date, there have been over a hundred canine cases and only two feline cases reported in the USA [2–4, 11, 17, 19–24, 26, 33, 35, 45–51]. A variety of factors may have also contributed to the higher prevalence found in this previous study when compared with ours [4], including the different anatomical location from where skin snips were collected f (e.g., nose versus interscapular region), a more favorable climate, and habitats that were less anthropogenically modified in comparison with the urban environment of Albuquerque. The lifestyle of dogs and cats on the reservation, which may include spending more time outdoors and the ability to roam free, may also have increased their likelihood for exposure to the bite of susceptible vectors. In addition, coyotes (*Canis latrans*) from Arizona and New Mexico are known to serve as a wild reservoir for *O. lupi* and are well-adapted to urban environments across their range in North America [17].

Outside North America, a few epidemiological studies have been conducted in *O. lupi*-endemic countries in southern Europe [15, 52], all of which have focused on dogs. In Greece and Portugal, prevalences of 8.7% (*n = *2/23) and 8.3% (*n = *7/84) were found, respectively, in a mixed population of client-owned and stray dogs. In southwestern Spain, the reported prevalence of *O. lupi* was slightly lower at 4.8% (*n = *5/104) in a shelter dog population [52]. There are several factors contributing to the varying prevalence documented in endemic areas across different countries. As previously discussed, some of these factors include differing sampling methodology, lifestyle of dog populations, presence or absence of clinical disease, as well as vector-associated and climate-associated parameters.

Although much remains to be elucidated regarding the biology of *O. lupi*, it has been generally assumed that its pre-patent period (PPP) is similar to that of other species of *Onchocerca*, ranging from 12 to 18 months [53, 54]. However, in the present study, two out of eight dogs were approximately 1 year old or younger. This finding suggests that *O. lupi* could have a shorter PPP than formerly postulated [24]. As the ages of the dogs enrolled in the study were estimated by veterinary professionals and not exact ages, controlled studies involving experimental infections of dogs of a known age would be required to fully elucidate this life cycle parameter. Nevertheless, it is known that *O. lupi* has a long lifespan, and possibly patency, estimated to be 3–8 years on the basis of case reports of dogs adopted from endemic regions and brought to nonendemic countries [24, 44, 54].

The novel probe-based qPCR utilized in this study was adapted from a previously published protocol to detect *O. lupi* DNA in hosts and putative vectors [27]. No genetic differences have been reported within the *cox1* region, as well as other mitochondrial and ribosomal regions of samples from the USA [3, 4, 17, 19–22, 44]. Our study presents a modification within the forward primer to detect the only genetic lineage of *O. lupi* reported to date across the USA, including the sequences generated in the current study [3, 4, 17, 20, 21, 34]. The implementation of this qPCR protocol in broader studies can inform on the potential establishment of this zoonotic parasite also in nonendemic regions of the USA, as there is limited veterinary and public health awareness regarding *O. lupi* across the country [54–56].

*Onchocerca lupi* is not the only filarioid nematode that can be present within the canine skin [29]. Within this study, we detected 20 dogs that were positive for *C. bainae* using cPCR. *Cercopithifilaria bainae* is transmitted by the brown dog tick, *Rhipicephalus sanguineus* s.l., which is found virtually worldwide [57–59]. To the author’s knowledge, there are only three known studies on *C. bainae* in the USA [60–62]. Two studies have been completed outside the USA that focus on screening for *Cercopithifilaria* spp. in skin samples and ticks [63, 64]. A large-scale epidemiological study that collected canine skin samples (*n = *917) and ticks (*n = *890) from Italy, Greece, and Spain found *C. bainae* in 13.9% and 10.5% using microscopy and cPCR, respectively [63]. Another study sampled five species of *Rhipicephalus* (i.e., *R. sanguineus* s.l., *R. turanicus*, *Rhipicephalus* sp. I, *Rhipicephalus* sp. II, and *Rhipicephalus* sp. III) collected from all over the world, from which *Cercopithifilaria* spp. were confirmed in 21.5% of samples via sequencing [64]. Although not the focus of our study, we found epidemiological data that showed *C. bainae* was present within approximately 5% of this study population. Although a previous study completed in the USA detected DNA within brown dog ticks collected from dogs in New Mexico, the current study is the first report of *C. bainae* in dogs themselves [62]. Since *O. lupi* and *C. bainae* have the potential to present as co-infections, it would be beneficial to design and validate a multiplex qPCR for both parasites, which could be implemented in future epidemiological investigations. To date, other *Cercopithifilaria* species that have been reported to infect domestic dogs (i.e., *Cercopithifilaria* sp. II and *Cercopithifilaria grassii*) have not been reported in the USA [65].

We did not find any significant risk factors associated with *O. lupi* infection in the studied canine population. A previous study focusing on clinical cases assessed risk factors potentially associated with *O. lupi* infection [51]. However, the authors excluded dogs with a mixed coat color and analyzed only those with a single coat color (e.g., black, brown, or white). The authors reported that large dogs, weighing more than 13.61 kg, with a single coat color, were at a higher risk of being infected with *O. lupi* when compared with small white and brown dogs [51]. Of the eight positive dogs found within our study, three were recorded as having a single-colored coat (i.e., black, tan, and yellow). Although we did not find similar evidence in the present study, it is important that we further assess risk factors, including those potentially associated with nonclinical *O. lupi* infection.

Diagnosing *O. lupi* in a clinical setting can be challenging, especially when veterinary professionals are unfamiliar with the parasite and, if present, the clinical signs associated with infection [3, 21, 66]. When subclinical infections are suspected, using microscopy combined with cPCR would be ideal; however, it requires a high level of training and reliance on a diagnostic laboratory that can perform a confirmatory molecular test. Therefore, the availability of a robust *O. lupi*-specific molecular diagnostic test for clinical use brings numerous benefits to the veterinary community, including rapid results, increased accuracy and precision, improved diagnostic sensitivity and specificity, extensive standardization of assays, and the potential for multiple pathogen screening [67–69]. A disadvantage of using DNA-based molecular techniques, such as qPCR, is the inability to determine whether microfilariae present within the host are dead or alive, making them unsuitable for confirming the infection status of an animal following treatment. For such cases, integrating classical microscopy for microfilariae isolation and morphological identification remains informative and necessary.

## Conclusions

This is the first epidemiological investigation on *O. lupi* in shelter dogs from an urban center in the Southwestern USA. This study determined a nearly 2% prevalence of *O. lupi* in dogs that would have likely gone undetected without the use of this novel qPCR. With the continued development of molecular diagnostic assays, we can further our understanding of biological parameters that remain unknown, leading to improvements in surveillance and the implementation of control strategies for this zoonotic parasite.

## Supplementary Information

Supplementary Material 1.

## Data Availability

Data supporting the main conclusions of this study are included in the manuscript.
