# Supplementary material for: Prevalence of Onchocerca lupi in shelter dogs from an endemic region of the Southwestern USA
Source: Parasit Vectors. 2025 Aug 5;18:335. doi: 10.1186/s13071-025-06988-5 (PMC12326697; doi:10.1186/s13071-025-06988-5)
Supplement: Supplementary file 1 — Supplementary Material 1. [file 13071_2025_6988_MOESM1_ESM.docx]

**
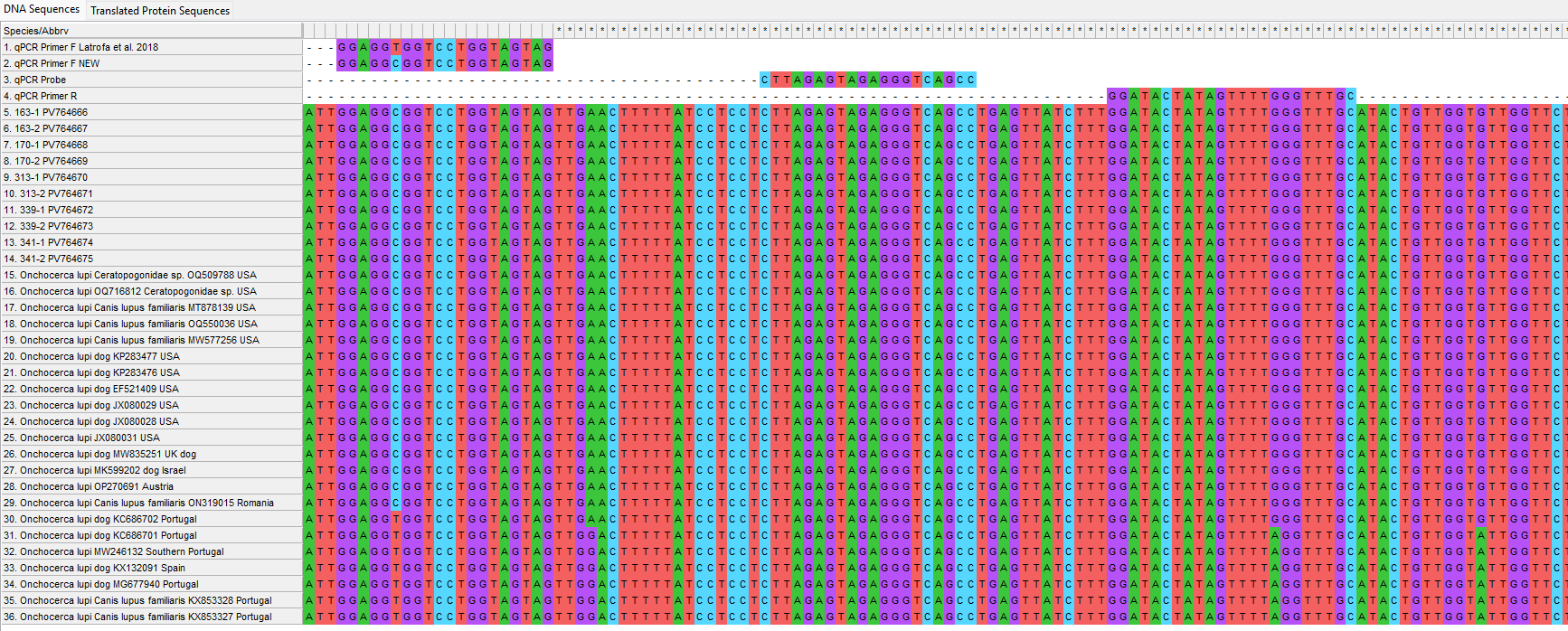
Supplementary Data, Figure S1.** A visual representation of *Onchocerca lupi* sequences at the cytochrome c oxidase subunit 1 highlight the genetic lineage present in USA, and those reported worldwide, showing a single nucleotide polymorphism within the forward primer sequence.

**Supplementary Data, Figure S2.** Standard curve of qPCR of detection for VetMAX^™^ Xeno^™^. This curve was created by plotting average CT values of three replicates using a 10-fold serial dilution of internal positive control DNA.
